# Supplementary material for: Effects of Hydrogen Sulfide on Carbohydrate Metabolism in Obese Type 2 Diabetic Rats
Source: Molecules. 2019 Jan 6;24(1):190. doi: 10.3390/molecules24010190 (PMC6337247; doi:10.3390/molecules24010190)
Supplement: Supplementary file 1 [file molecules-24-00190-s001.pdf]

Supplementary Materials

Effects of hydrogen sulfide on carbohydrate metabolism in obese type 2 diabetic rats

Sevda Gheibi[1], Sajad Jeddi[1], Khosrow Kashfi[2,*]Asghar Ghasemi[1,*]

1. Endocrine Physiology Research Center, Research institute for Endocrine Sciences, Shahid Beheshti University of Medical Sciences, Tehran, Iran
2. Department of Molecular, Cellular and Biomedical Sciences, Sophie Davis School of Biomedical Education, City University of New York School of Medicine, New York, USA

E-mail address: Ghasemi@endocrine.ac.ir

Email: kashfi@med.cuny.edu

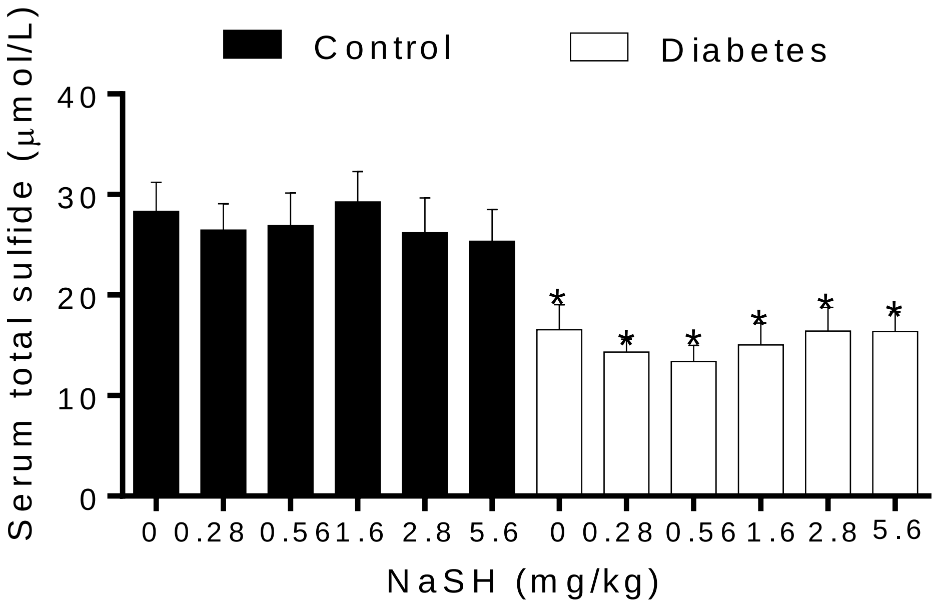

Figure S1. Serum total sulfide levels before NaSH administration. *Significantly different compared to non-treated control group (P<0.05). Values are mean ± SEM (n=10).

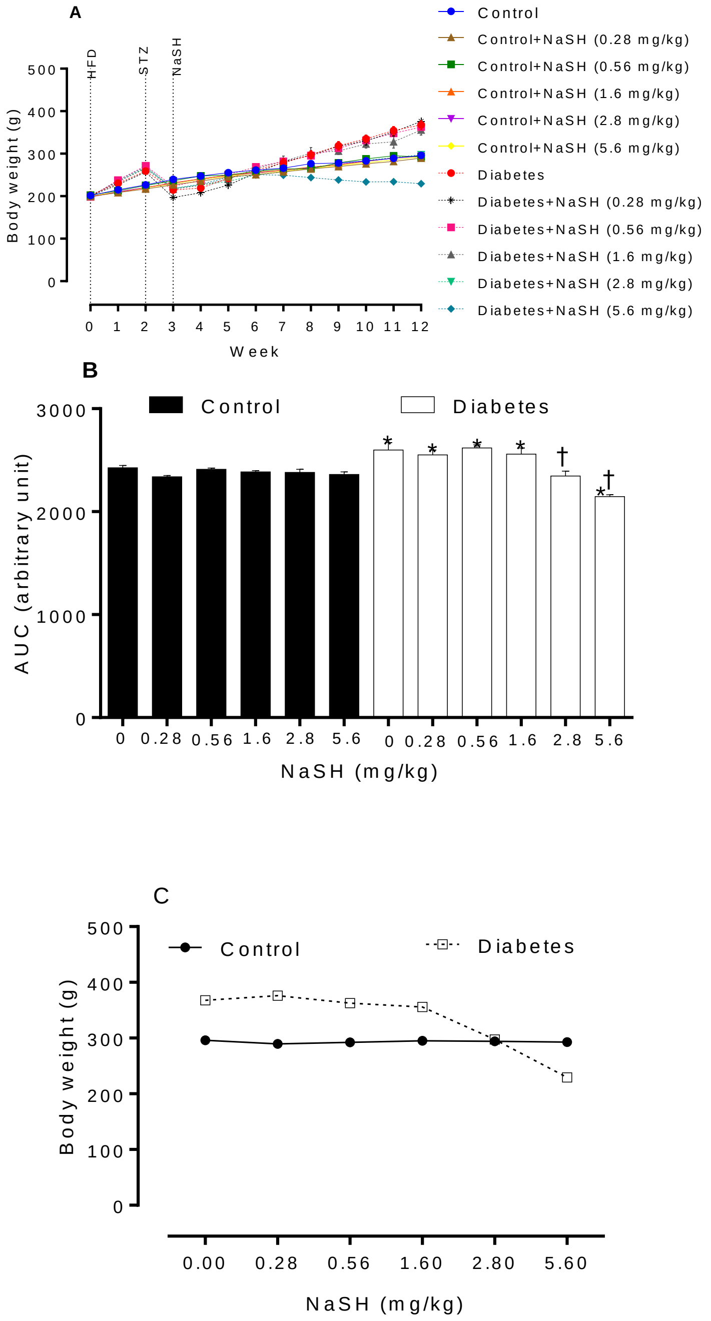

Figure S2. Effects of NaSH on body weight (A). Area under the curves (from week 3 to week 12) are shown in B and the relation between dose of NaSH and effect is shown in C. * Statistically significant difference compared to untreated control group. † Statistically significant difference compared to un-treated diabetic group. Values are mean ± SEM. (n=10). HFD, start of high fat diet; STZ, STZ injection; NaSH, start of NaSH injection.

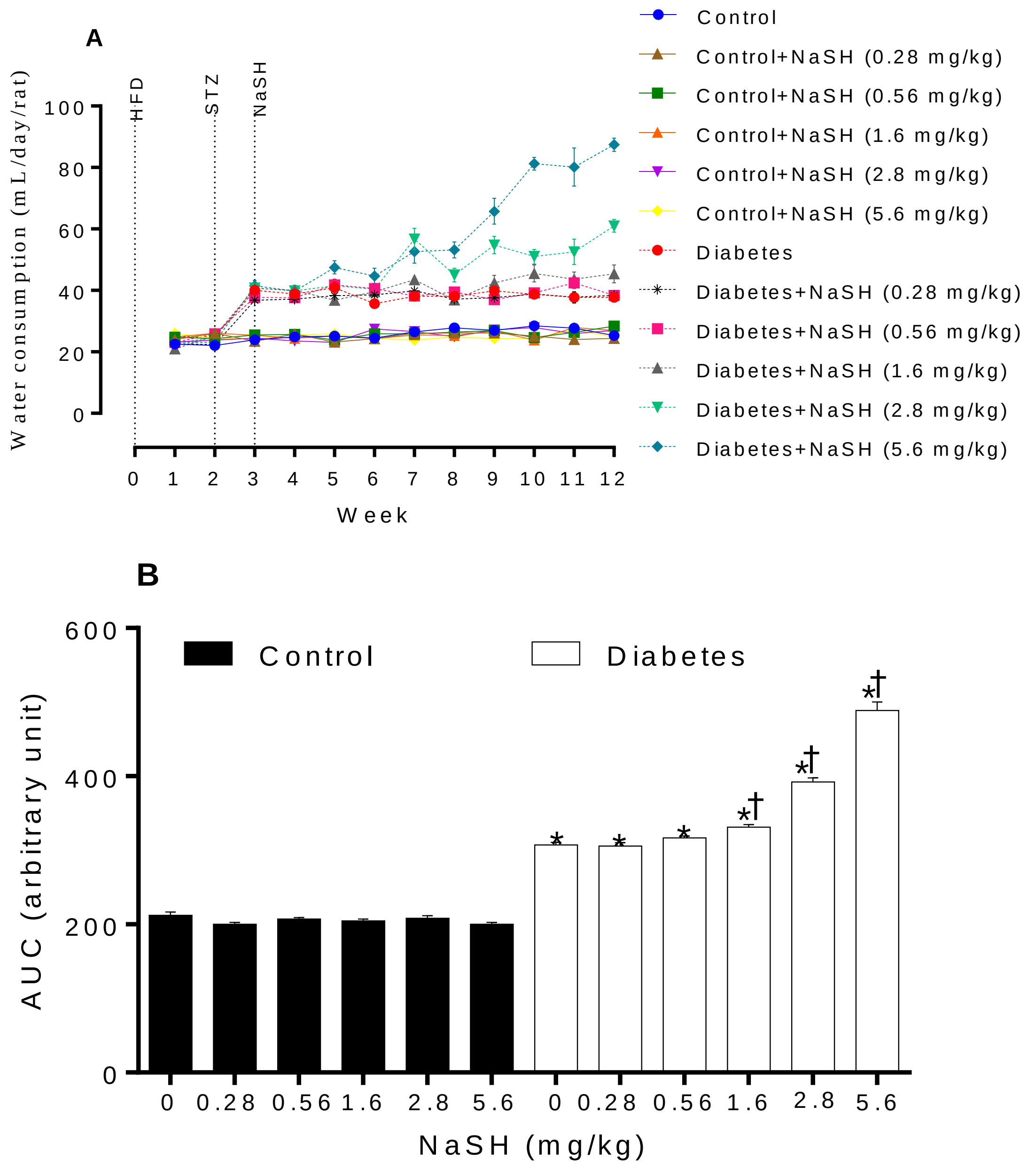

Figure S3. Effects of NaSH on water consumption (A). Area under the curves (from week 3 to week 12) are shown in B. * Statistically significant difference compared to un-treated control group. † Statistically significant difference compared to un-treated diabetic group. Values are mean ± SEM. (n=10). HFD, start of high fat diet; STZ, STZ injection; NaSH, start of NaSH injection.

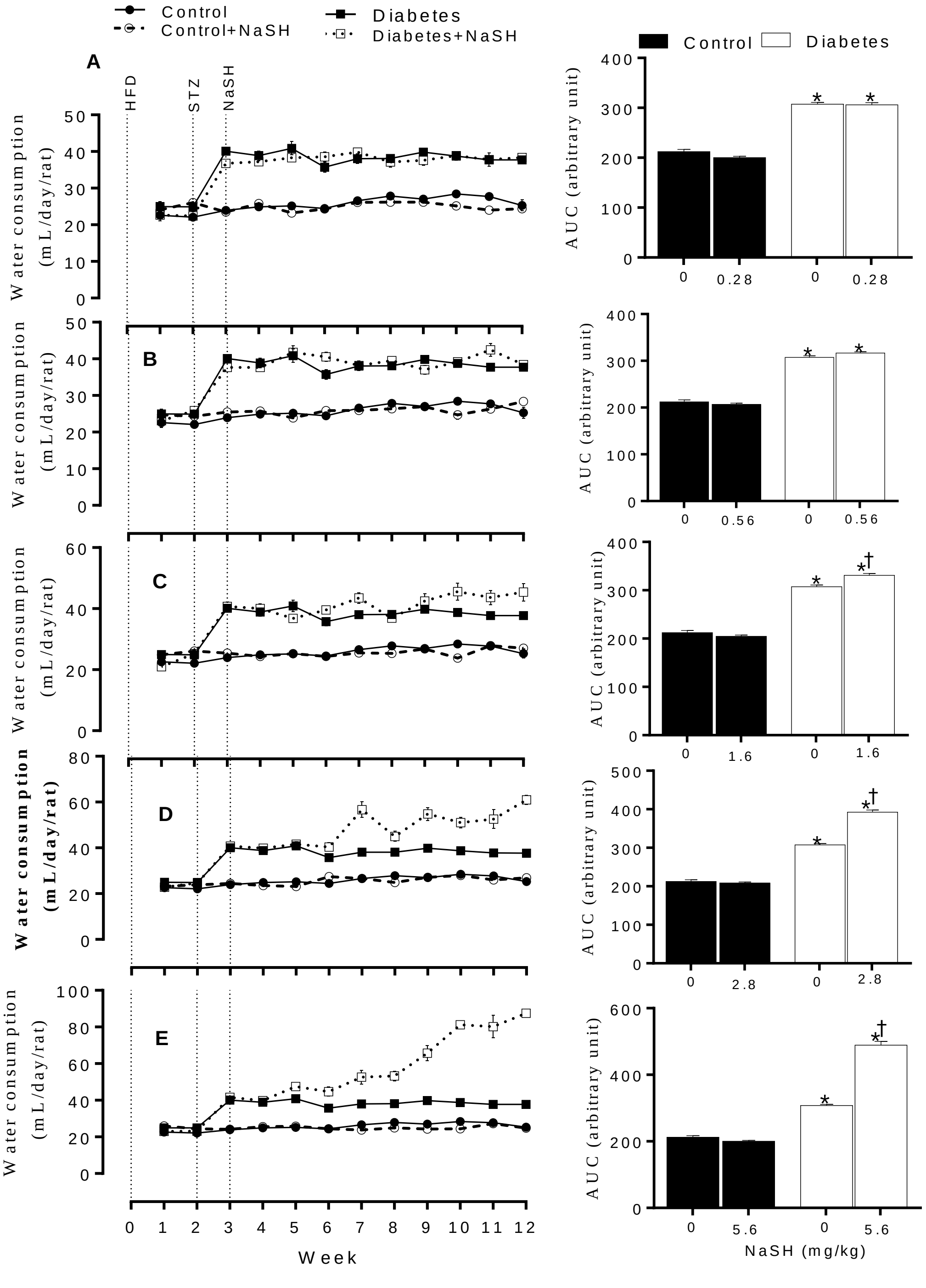

Figure S4. Effects of NaSH on water consumption at 0.28 (A), 0.56 (B), 1.6 (C), 2.8 (D), and 5.6 (E) mg/kg. Area under the curves (from week 3 to week 12) are shown in columns on the right. * Statistically significant difference compared to un-treated control group. † Statistically significant difference compared to un-treated diabetic group. Values are mean ± SEM. (n=10). HFD, start of high fat diet; STZ, STZ injection; NaSH, start of NaSH injection.

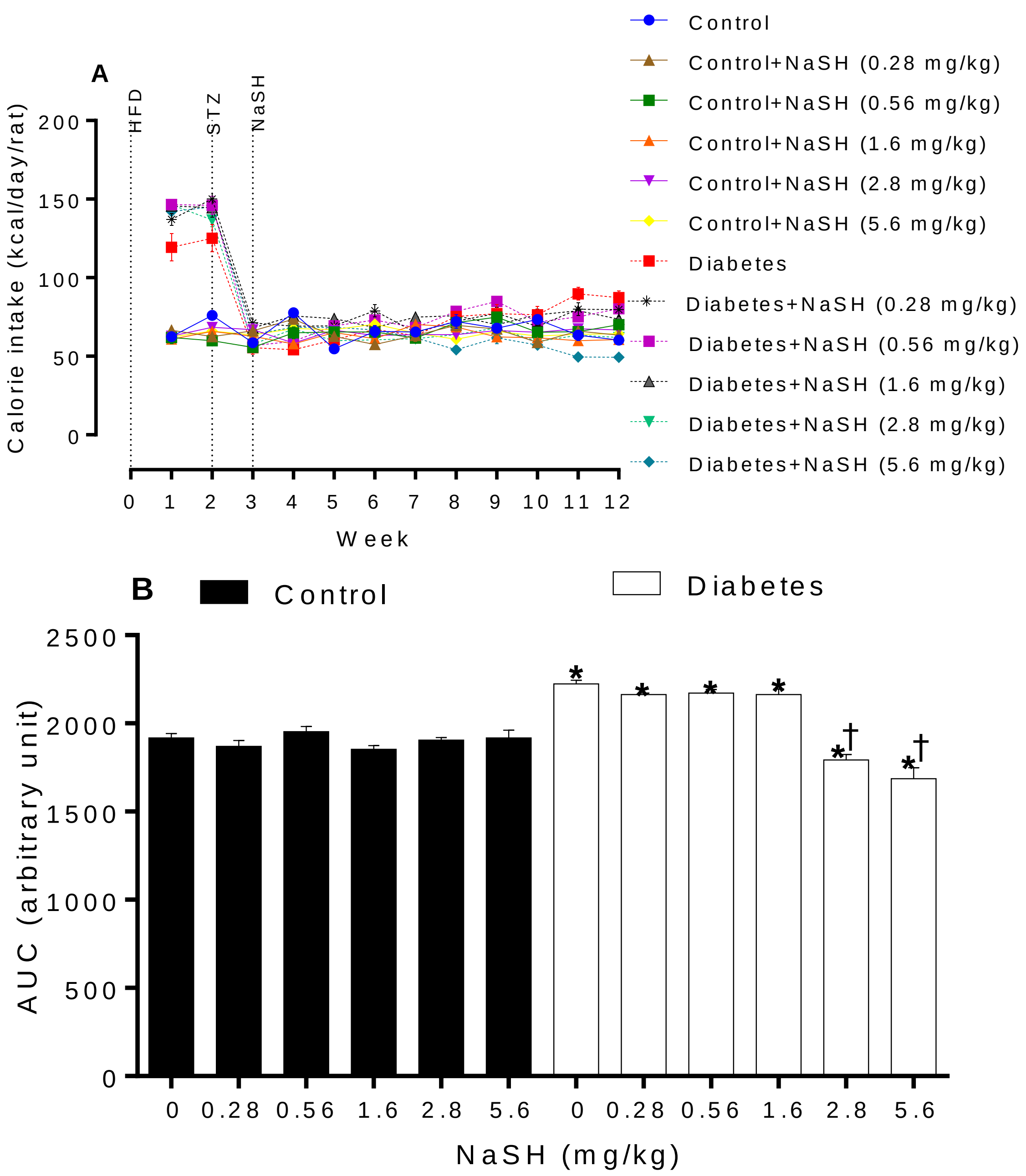

Figure S5. Effects of NaSH on calorie intake (A). Area under the curves (from week 3 to week 12) are shown in B. * Statistically significant difference compared to un-treated control group. † Statistically significant difference compared to un-treated diabetic group. Values are mean ± SEM. (n=10). HFD, start of high fat diet; STZ, STZ injection; NaSH, start of NaSH injection.

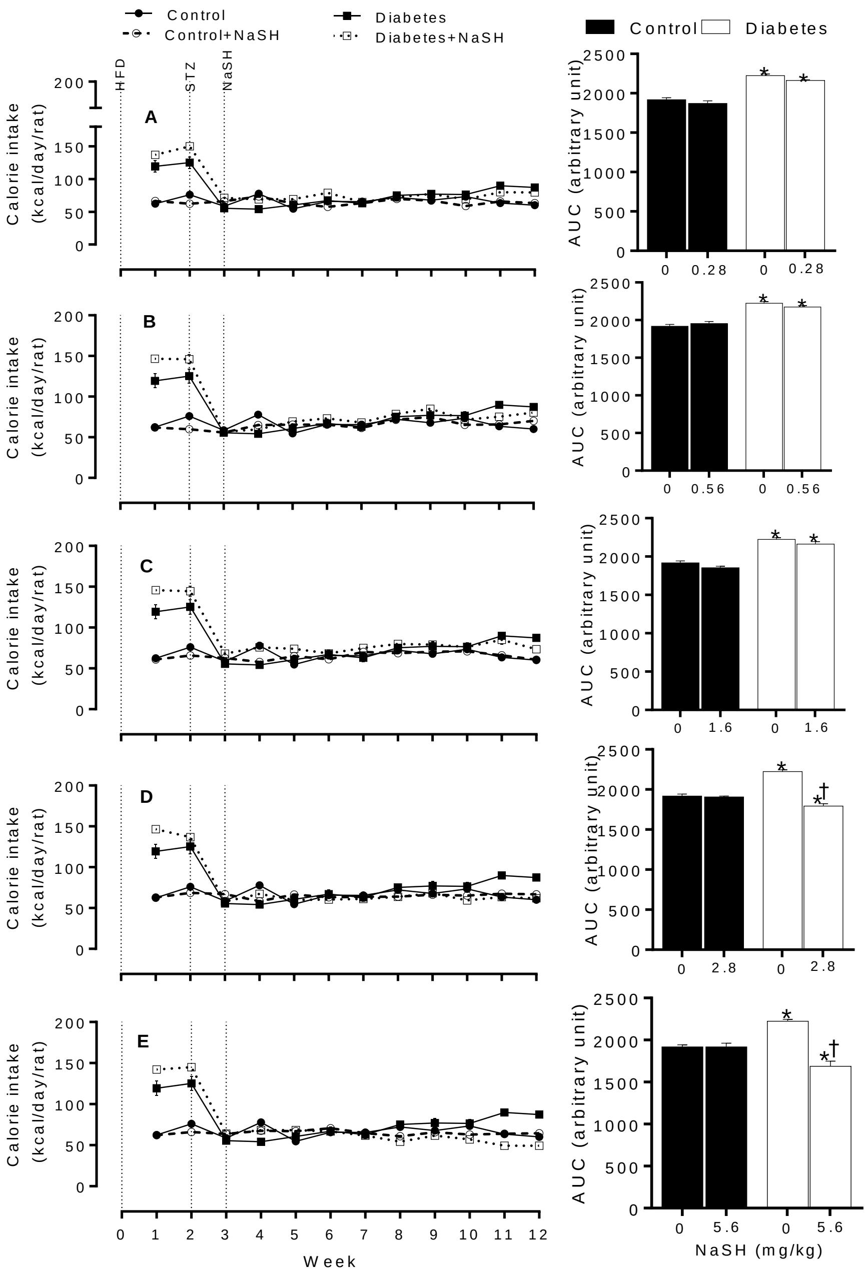

Figure S6. Effects of NaSH on calorie intake at 0.28 (A), 0.56 (B), 1.6 (C), 2.8 (D), and 5.6 (E) mg/kg. Area under the curves (from week 3 to week 12) are shown in columns on the right. * Statistically significant difference compared to un-treated control group. † Statistically significant difference compared to un-treated diabetic group. Values are mean ± SEM. (n=10). HFD, start of high fat diet; STZ, STZ injection; NaSH, start of NaSH injection.

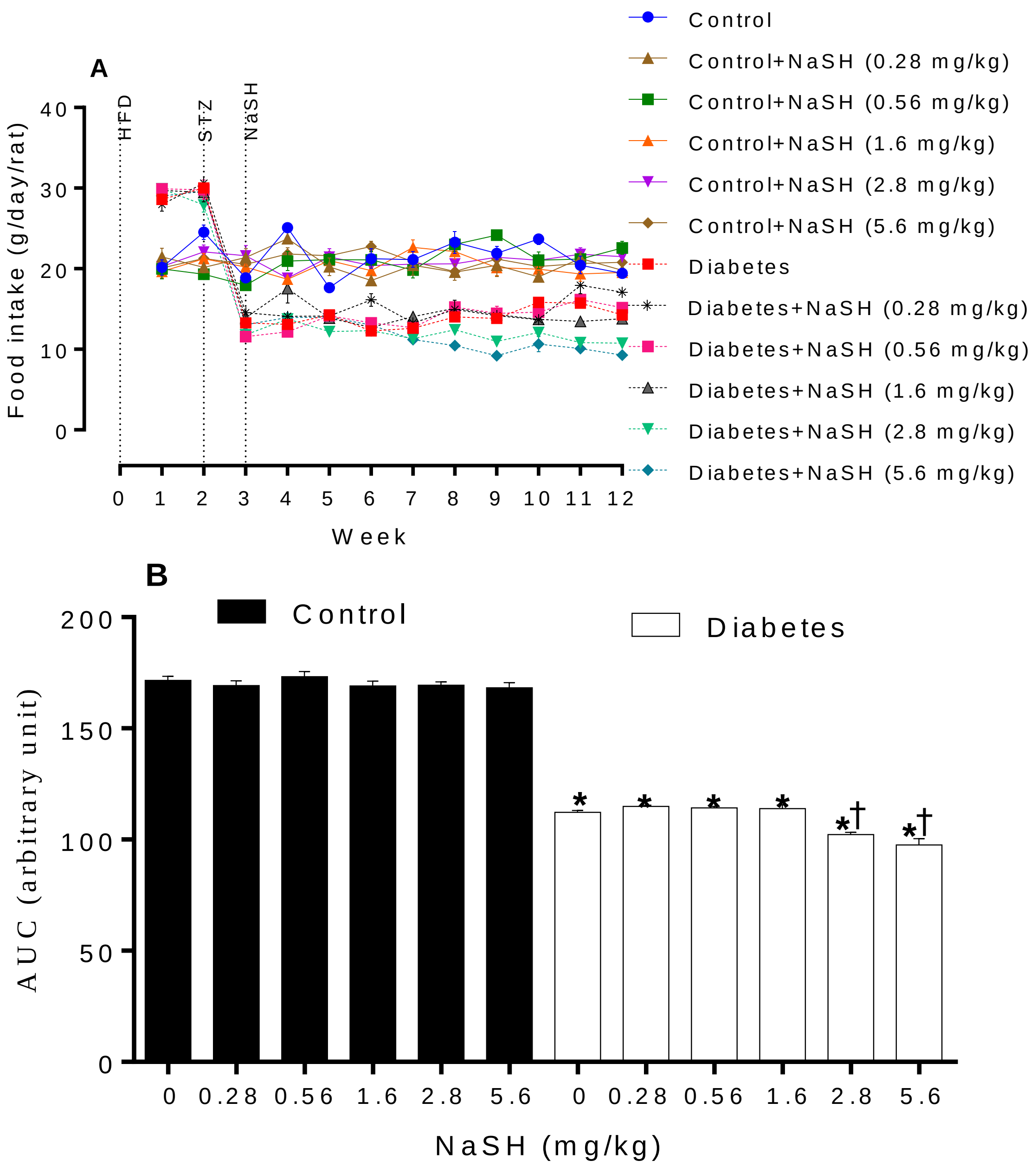

Figure S7. Effects of NaSH on food intake (A). Area under the curves (from week 3 to week 12) are shown in B. * statistically significant difference compared to un-treated control group. † Statistically significant difference compared to un-treated diabetic group. Values are mean ± SEM. (n=10). HFD, start of high fat diet; STZ, STZ injection; NaSH, start of NaSH injection.

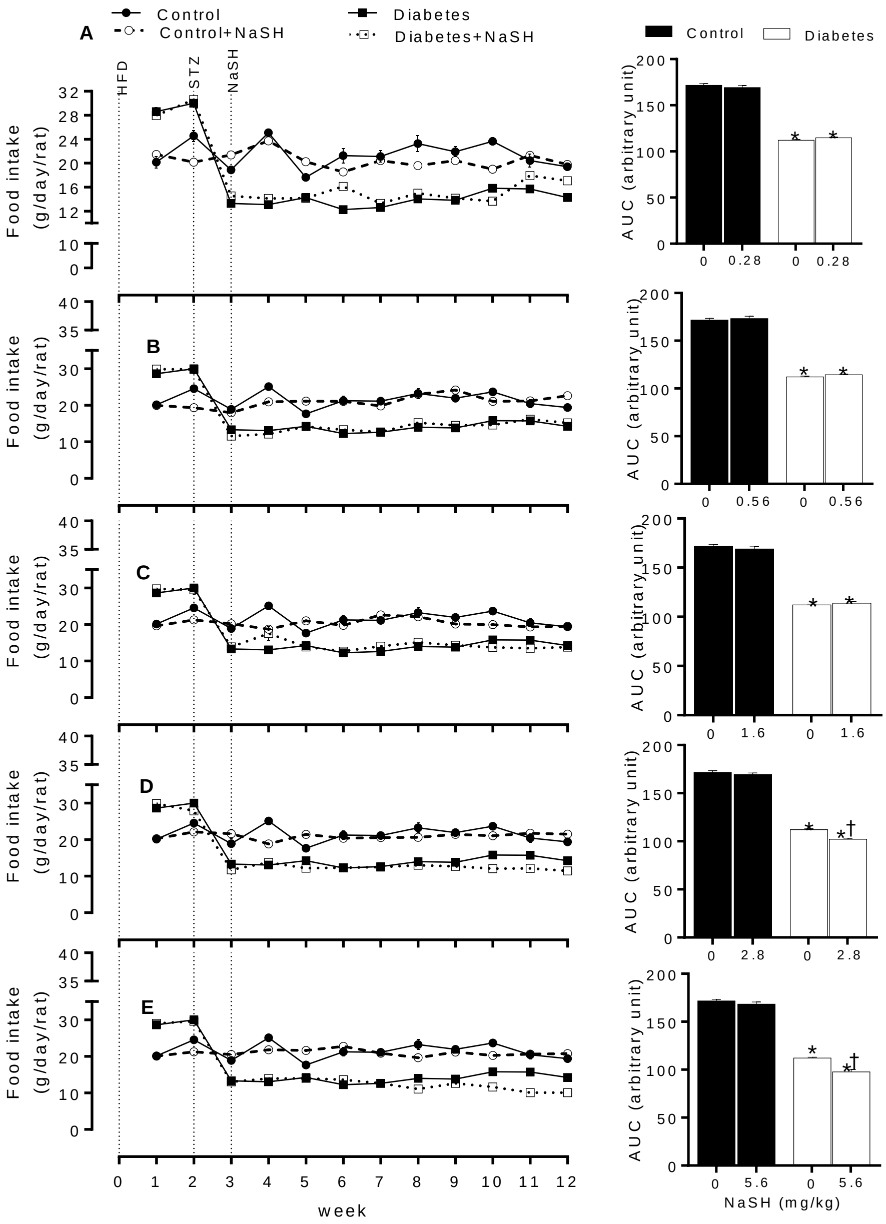

Figure S8. Effects of NaSH on food intake at 0.28 (A), 0.56 (B), 1.6 (C), 2.8 (D), and 5.6 (E) mg/kg. Area under the curves (from week 3 to week 12) are shown in columns on the right. * Statistically significant difference compared to un-treated control group. † Statistically significant difference compared to un-treated diabetic group. Values are mean ± SEM. (n=10). HFD, start of high fat diet; STZ, STZ injection; NaSH, start of NaSH injection.

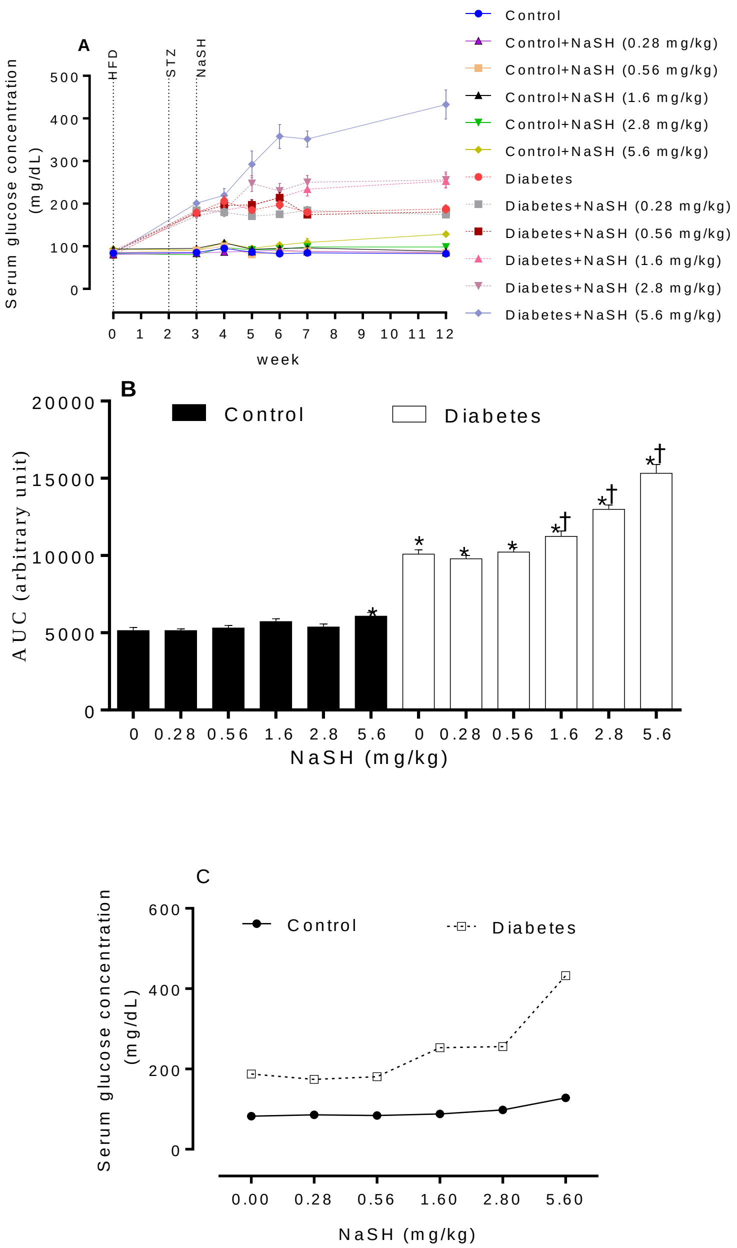

Figure S9. Effects of NaSH on serum glucose (A). Area under the curves (from week 3 to week 12) are shown in B and the relation between dose of NaSH and effect is shown in C. * Statistically significant difference compared to un-treated control group. † Statistically significant difference compared to un-treated diabetic group. Values are mean ± SEM. (n=10). HFD, start of high fat diet; STZ, STZ injection; NaSH, start of NaSH injection.

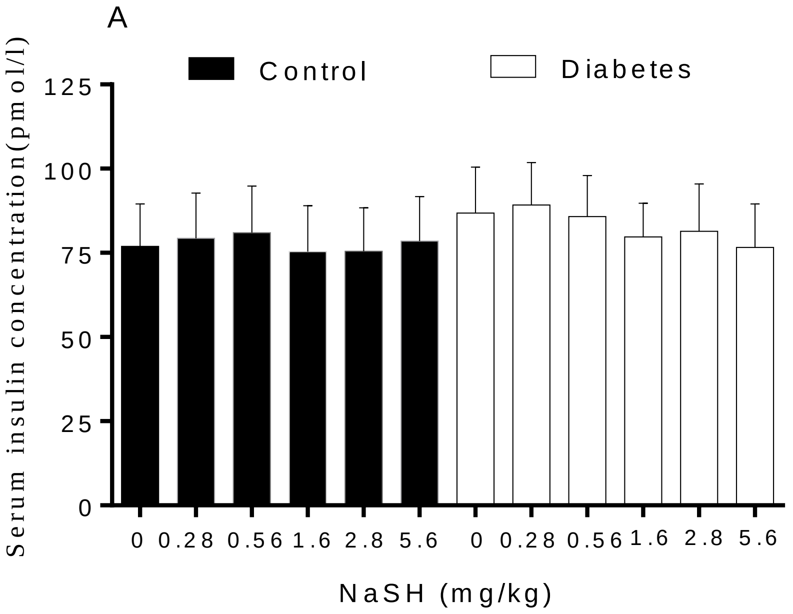

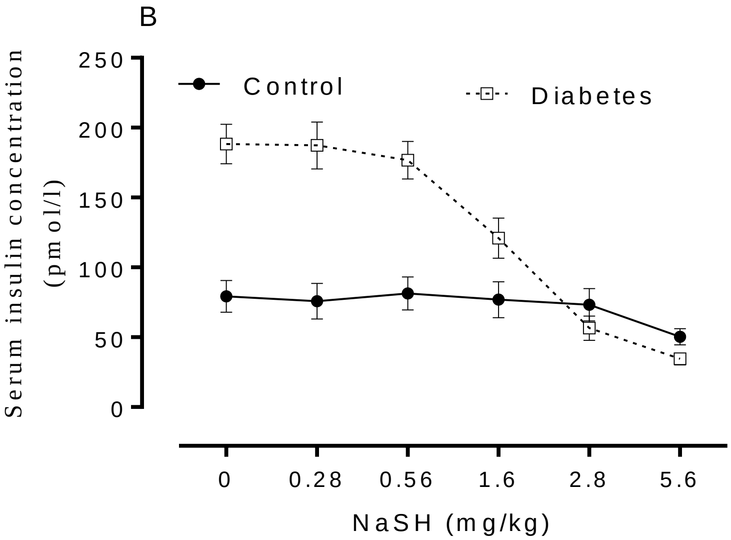

Figure S10. Serum insulin levels before NaSH administration (A). The relation between dose of NaSH and effect is shown in B. Values are mean ± SEM. (n=8).

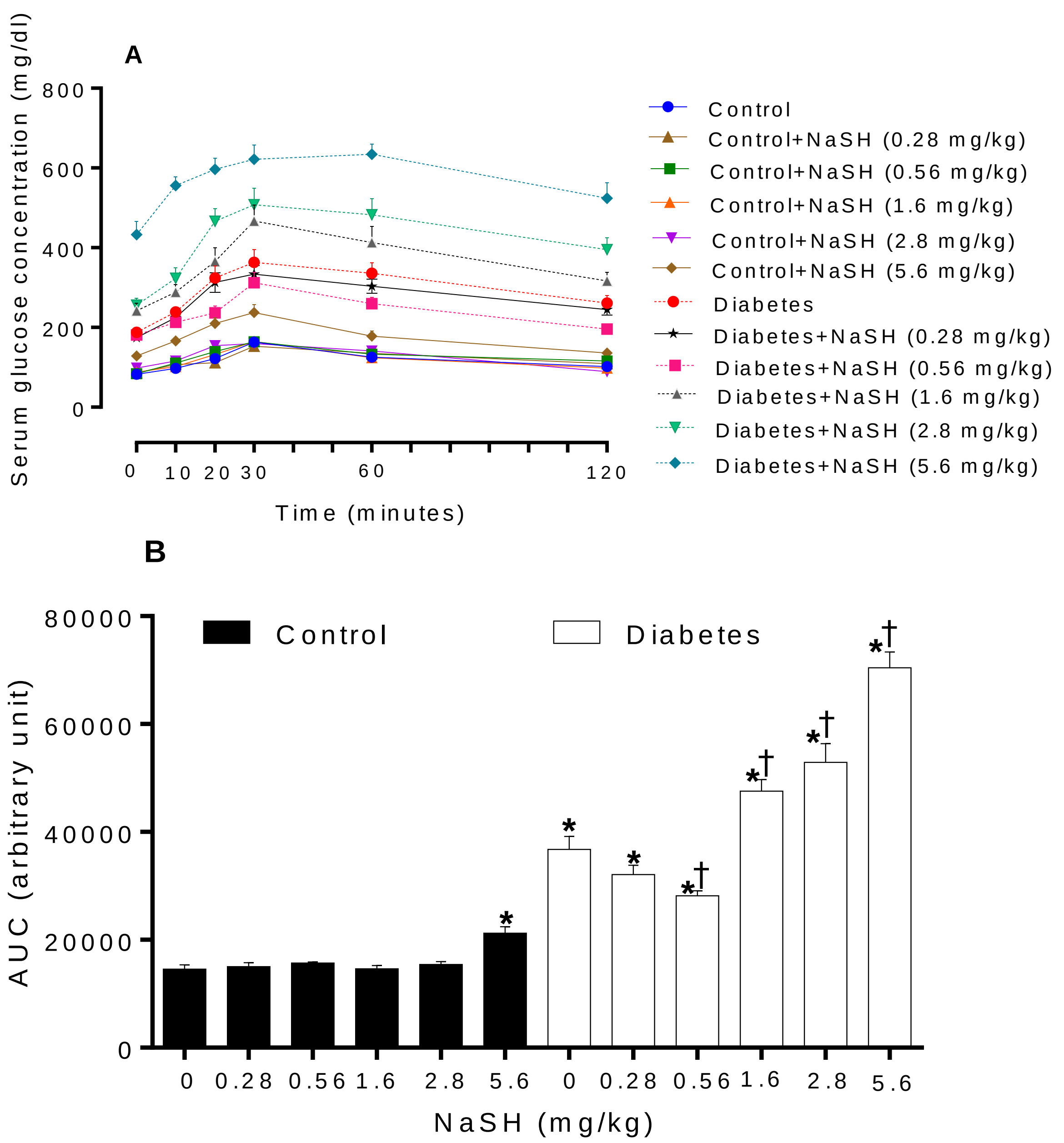

Figure S11. Effects of NaSH on glucose tolerance (A). Area under the curves are shown in B. * Statistically significant difference compared to un-treated control group. † Statistically significant difference compared to un-treated diabetic group. Values are mean ± SEM. (n=10).

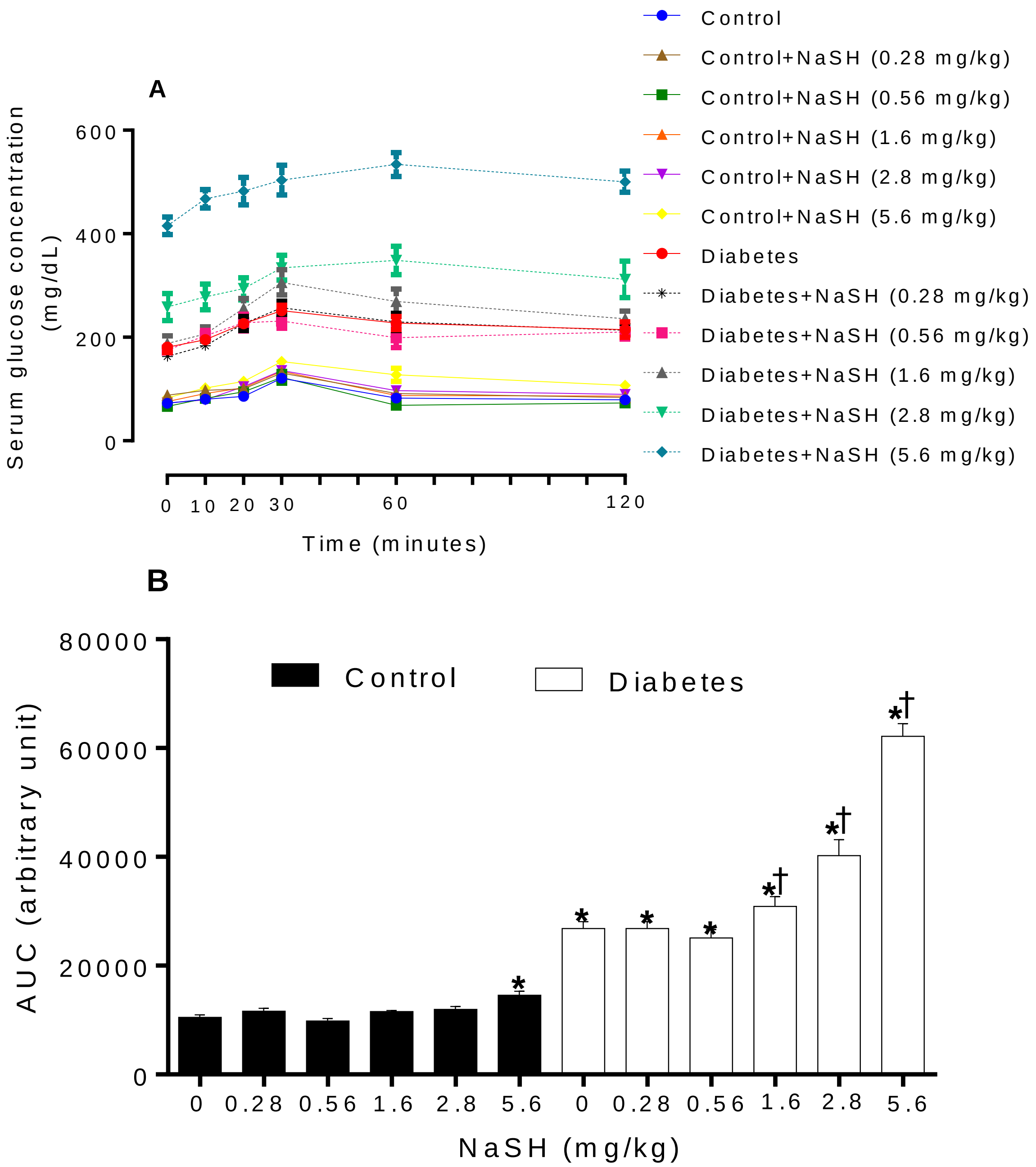

Figure S12. Effects of NaSH on pyruvate tolerance (A). Area under the curves are shown in B. * Statistically significant difference compared to un-treated control group. † Statistically significant difference compared to un-treated diabetic group. Values are mean ± SEM. (n=10).

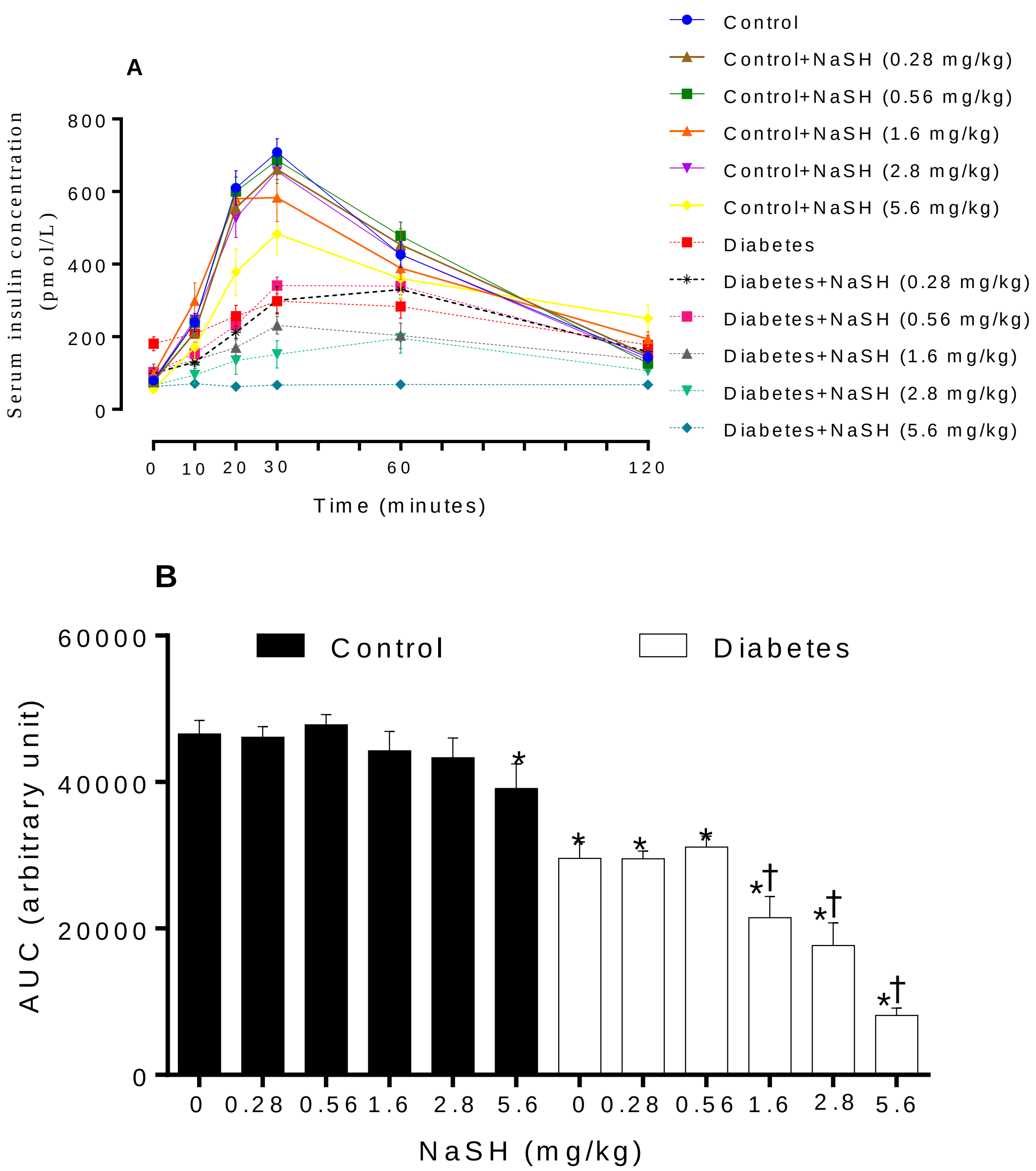

Figure S13. Effects of NaSH on *in vivo* insulin secretion (A). Area under the curves are shown in B. * Statistically significant difference compared to un-treated control group. † Statistically significant difference compared to un-treated diabetic group. Values are mean ± SEM. (n=8).
